# Spread of human cancer cells occurs with probabilities indicative of a nongenetic mechanism

**DOI:** 10.1038/sj.bjc.6602848

**Published:** 2005-11-08

**Authors:** J S Michaelson, J A Cheongsiatmoy, F Dewey, M J Silverstein, D Sgroi, B Smith, K K Tanabe

**Affiliations:** 1 Department of Pathology, Massachusetts General Hospital, Boston, MA, USA; 2 Department of Surgery, Massachusetts General Hospital, Boston, MA, USA; 3 Department of Surgery, Harvard Medical School, Boston, MA, USA; 4 Department of Pathology, Harvard Medical School, Boston, MA, USA; 5 Department of Surgery, Keck School of Medicine, University of Southern California, Los Angeles, CA, USA

**Keywords:** metastasis, probability, mutation

## Abstract

There has been much uncertainty as to whether metastasis requires mutation at the time of spread. Here, we use clinical data to calculate the probability of the spread of melanoma and breast cancer cells. These calculations reveal that the probability of the spread of cancer cells is relatively high for small tumours (∼1 event of spread for every 500 cells for melanomas of 0.1 mm) and declines as tumours increase in size (∼1 event of spread for every 10^8^ cells for melanomas of 12 mm). The probability of spread of breast cancer cells from the lymph nodes to the periphery is ∼1 event of spread for every 10^8^ cells in the nodal masses, which have a mean diameter of 5 mm, while the probability of spread of cancer cells from the breast to the periphery when the primary masses are 5 mm is also ∼1 event of spread for every 10^8^ cells. Thus, the occurrence of an event of spread from the breast to the lymph nodes appears not to increase the propensity of the progeny of those cells to spread from the lymph nodes to the periphery. These values indicate that the spread of human breast cancer and melanoma cells is unlikely to occur by a mechanism requiring mutation at the time of spread.

There has been much uncertainty as to whether metastasis requires mutation at the time of spread (Cifone and Fidler, 1981; Fidler, 1983; Sobel, 1990; Welch *et al*, 2000; Yokota, 2000; Bernards and Weinberg, 2002; Couzin, 2003; Bernards, 2003; Van't Veer and Weigelt, 2003; Yang *et al*, 2004). Mutations have a number of characteristic features, in terms of the rates of their occurrence and other qualities, which are diagnostic: mutations are rare, a phenotype conferred on a cell by mutation is inherited by the progeny of the cell, and the rates of the appearance of phenotypes caused by mutations either remains constant over time for those phenotypes requiring only a single mutation, or increase in frequency for those phenotypes requiring the accumulation of multiple mutations. We have recently shown that from clinical data it is possible to measure the rates of metastatic spread, expressed in terms of the probability of spread per cell (Michaelson, 1999; Michaelson *et al*, 2002, 2003). Here we use this methology to measure the probability of spread per cell for human breast cancer and melanoma. The values of these probabilities are inconsistent with metastasis occurring by a process of mutation.

## METHODS

### Data

Data on the relationship between tumour size and breast cancer survival is from the USC/Van Nuys population (Silverstein, 2000; Michaelson *et al*, 2002, 2003), from Tabar *et al* (2000) and Tubiana and colleagues (Koscielny *et al*, 1984; Tubiana and Koscielny, 1990, 1991). For details and the general equivalence of these survival values (based on the 15-year Kaplan–Meier survival rate, based upon Karrison *et al* (1999) finding that it is not until this point in time that the survival rate become clear), see Michaelson *et al* (2002, 2003). Data on the relationship between tumour thickness and melanoma survival are 10-year Kaplan–Meier disease survival values from Balch *et al*, 2001).

Tumour diameters (breast cancer) and thickness (melanoma) were taken at pathological analysis. Since we shall be interested in tumour size in terms of the number of cells that they contain, *N*, we can generate rough estimates of the value of *N* that are quite satisfactory for our purposes here by converting values of tumour diameter or thickness, *D*, into values of cell number, *N*, assuming spherical geometry and a density of *s* (here we shall use 10^8^ cells/cm^3^ as a biologically plausible estimate of, *s*, as outlined in Boon *et al*, 1982; Pesce and Colacino, 1986; Van der Linden *et al*, 1986; and Michaelson *et al*, 2002). While this estimate of the value of *s* is biologically reasonable, for the purposes of the calculations made here, it need not be precise, as any error in the estimation of *s* by an order of magnitude or more will not change the general lessons drawn here on the nature of cancer spread, although it will affect the precise values of the probability of spread.

Node-positive patients are defined as those patients with one or more lymph nodes found to have cancer upon pathological analysis. The range of nodes examined among women in the USC/Van Nuys populations varied from 1 to 47; however, only 10% of women had fewer than 10 nodes examined, while only 2% of women had fewer than five nodes examined. The mean and median number of nodes examined was 16.8 and 17, with a s.d. of 7.2.

Information on the size of the cancer metastases in the lymph nodes was collected from microscope slides from 16 node-positive patients, chosen at random, among MGH patients with invasive breast cancer diagnosed in 1993, among which there were 49 positive lymph nodes. Microscopic imagines of each node were captured with a Nikon Eclipse E400 microscope equipped with an Insight digital camera (Diagnostic Instruments Inc., Sterling Heights, MI, USA), used to capture high-resolution noninterpolated image with a × 10 objective. The image measurements were calibrated by comparison to an image of the 1 mm grating on a haemocytometer. The longest dimension of the metastasis was measured from prints made of the images. All slides were reviewed by a qualified breast pathologist (DS). In some nodal metastases, noncancerous cells were apparent within the mass of cancer, and the sizes of these cancerous and noncancerous areas were measured. Thus, the values labelled ‘Diameter (corrected)’ (Table 3) were corrected with respect to the cancerous component of each metastasis, and thus were representative of the size that a mass of cancer would have had, had it shown the same number of cells but no noncancerous component.

### Mathematical methods

Following the line of thinking outlined previously (Michaelson *et al*, 2002), let us define *p* as the probability of a single successful event of metastatic spread prior to surgery per unit of tumour volume, *s*. When the value of *s* is chosen so as to be to be equivalent to the volume of a cell, then *p*, from a practical standpoint, is also the probability of spread per cell, *N*. Note that by defining *p* on a per volume or per-cell basis, we are not assuming that every cell in the tumour has the potential to spread. For example, if we find that in a specific context, *p*=1-in-ten-billion, then we shall not mean that every cell in the tumour mass will have such a chance of spread. Rather, this simply means that for every ten billion cells in a tumour, there will be about one event of metastatic spread. Note also that we have defined *p* as the probability of an *event* of spread, which can be either the spread of a single cell or a cluster of cells. Additionally, since we are defining *p* in terms of *successful* events of spread, that is events of spread that go on to give rise to evident cancer in the local nodes, or to give rise to distant metastatic disease, we are not concerned with those events of spread that do not result in such manifestations of metastasis. Let us define *L* as the fraction of patients displaying the occurrence of such an event of spread. If we are interested in examining the probability of the lethal spread of cells to the periphery, resulting in metastatic disease, then *L* will be the fraction of patients dying of the cancers; while if we are interested in measuring the nonlethal spread of cancer cells to the lymph nodes, then *L* will be the fraction of patients with cancer found in the nodes upon pathological analysis. It follows that (1-*L*) will be the fraction of the fraction of patients *not* displaying the occurrence of spread Similarly, as *p* is the per-cell probability of an event of spread, the probability that there will *not* be an event of spread will be (1-*p*), and the overall probability that a tumour of *N* cells has not given rise to one or more such metastases will be (1-*p*)^*N*^. It has long been appreciated that for small values of *p*, (1-*p*)^*N*^ can very well be approximated by e^*-Np*^, and thus: 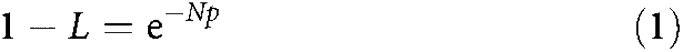


Rearranging provides a way to estimate the probability of spread (*p*) per cell (*N*): 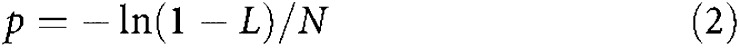


## RESULTS

From clinical data, we are able to observe the consequences of several examples of the spread of cancer cells, and their probabilities: the lethal spread of breast cancer and melanoma cells from the primary site to the periphery (*p*_BC-overall_ and *p*_MEL-overall_); and the nonlethal spread of breast cancer cells from the primary site to the local nodes (*p*_BC-to-nodes_), and the lethal spread of breast cancer cells from the lymph nodes to the periphery (*p*_BC-from-nodes_) (Tables 1 and 2). To see the general approach for estimating the values of these probabilities, consider the simple example of a group of patients with tumours containing a billion cells (*N*=10^9^ cells, ∼3 cm), of whom 10% have died of metastatic disease (*L*=0.1). If we assume, for explanatory purposes, that each death was the result of the spread of a single cell from the primary site to the periphery (an assumption not made in the math outlined in equations (1) and (2) above) then it follows that the probability (*p*) of lethal spread is approximately 1 event of spread for every ten billion cells in the primary mass (*p*≈*L/N*=0.1/10^9^). Similarly, if about 1% patients with a different type of tumour, but of the same size (*N*=10^9^ cells), have died of metastatic disease (*L*=0.01), then it follows that the probability of lethal spread (*p*) is about 1 event of spread for every hundred billion cells (*p≈L/N*=0.01/10^9^).

**Figure 1:**
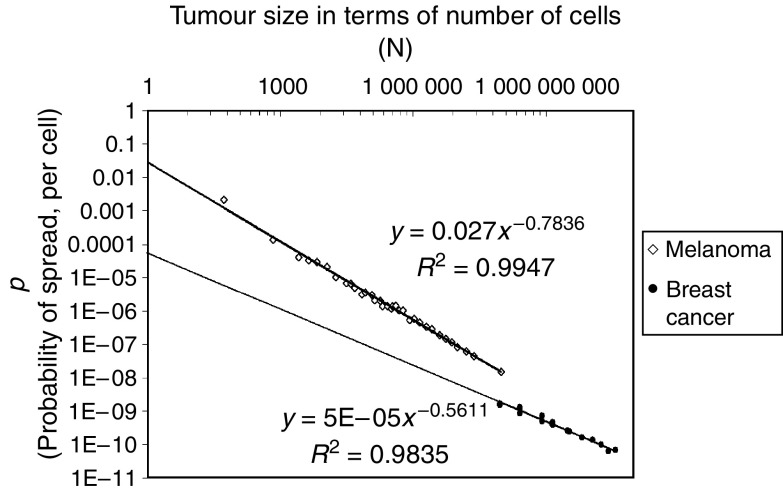
Calculations of the probability of lethal spread of breast cancer and melanoma cells, as a function of tumour size, and the close fit of the data to equation (2). (*R*^2^=0.98 for breast cancer, (*R*^2^=0.9 for melanoma). Shown here are the overall values for the probability of lethal spread of cancer cells from the primary site to the periphery for breast cancer (*p*_BC-overall_) and melanoma (*p*_MEL-overall_) using tumour size/survival data for all patients (Table 1). Note the close fit to the power function, equation (3).

**Figure 2:**
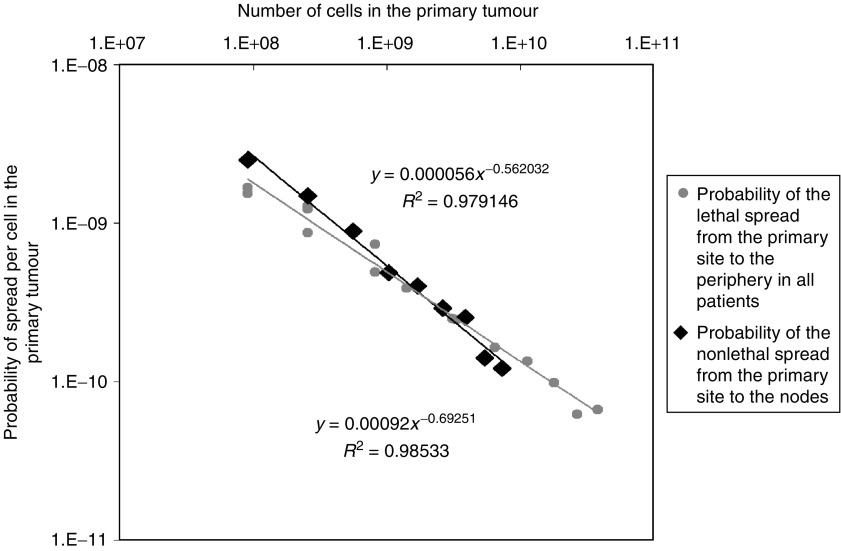
Calculations of the probability of lethal spread of breast cancer from the primary site to the periphery (*p*_BC-overall_) by equation (2) and using tumour size/survival data for all patients (Table 1), and the probability of nonlethal spread of breast cancer from the primary site to the lymph nodes (*p*_BC-to-nodes_) by equation (2) and using tumour size/nodal status data (Table 1). Note that in both cases the relationship between the probability of spread and tumour size is well fit by a power function, equation (3).

Equation (2) provides the technique for accurately quantifying the probabilities of these types of cancer spread. In three instances (*p*_BC-overall_, *p*_MEL-overall_ and *p*_BC-to-nodes_, Tables 1 and 2), data are available on spread from tumours of various sizes, revealing that the probability values do not remain constant as tumours grow, but declines as tumours increase in size (Figures 1 and 2). For example, the probability of lethal spread of melanoma cells from the primary site in the skin to the periphery (*p*_MEL-overall_) is ∼1 event of spread for every 500 cells for melanomas of 0.1 mm, but 100,000-fold lower (∼1 event of spread for every 10^8^ cells) for 12 mm tumours (Figure 1). As we have reported previously (Michaelson *et al*, 2002), a similar decline in the probability of spread per cell is also seen as tumours become larger for the overall probability of lethal spread of breast cancer cells from the primary site in the breast to the periphery (*p*_BC-overall,_ Figure 1). As can also be seen in Figure 1, a similar decline in the probability of spread per cell occurs for the nonlethal spread of breast cancer cells from the primary site in the breast to the lymph nodes (*p*_BC-to-nodes,_ Figure 2) (Tables 1 and 2). Furthermore, in each of the three contexts this decline occurs in a highly predictable fashion with *N*, such that it is well fit (Figures 1 and 2) to a power function of the form: 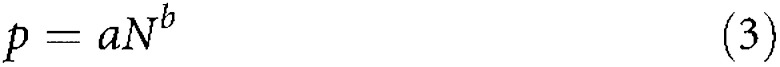


Values for *a* and *b* for each of these three types of metastatic spread are shown in Table 2. *b* has a negative value of approximately −0.5 to −0.8, reflecting the fact that the value of *p* declines as tumours increase in size. *a* can be thought of as the probability of spread for the very first cell in the tumour, because *p*=*a* when *N*=*1*. Note that the parameter *a* is approximately 500-fold higher for melanoma than for breast cancer, reflecting the long-appreciated greater propensity of melanoma to give rise to metastases.

**Figure 3:**
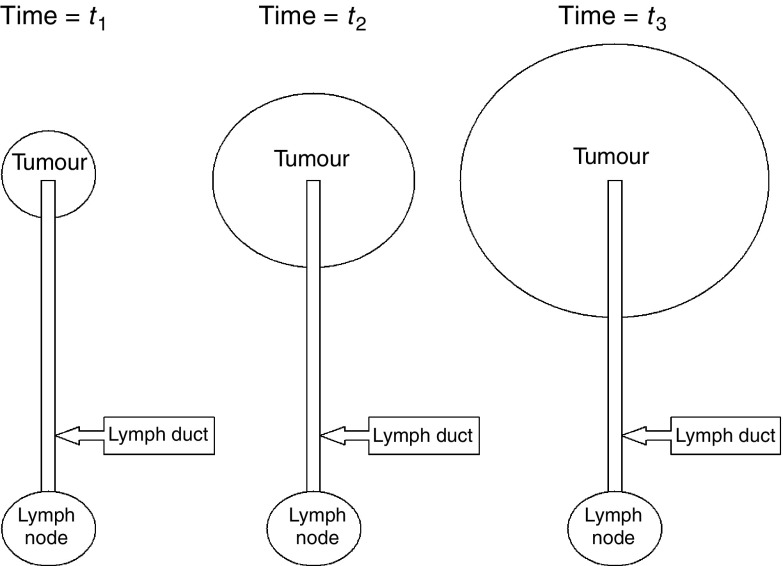
Schematic of Geometrical Model #1. Shown is a highly idealised image of a tumour mass and a lymph duct leading to a local lymph node.

There are a number of possible explanations for why the probability of spread per cell, *p*, declines as tumours become larger. It could be that only a subpopulation of tumour cells are capable of metastasising, and that the relative abundance of these cells decline as tumours grow. Another possibility is that the decline in the per-cell probability of spread is the result of the simple geometrical constraints posed to the escape of cells from the primary mass (Padera *et al*, 2002), which become more formidable as tumours increase in size. As shown in the Supplementary material, such possibilities are mathematically possible, and are testable in experimental systems (Figure 3).

The spread of cancer cells can occur in single steps, such as the spread of a cell directly from the primary site to the periphery, or in multiple steps, such as the initial spread of a cell from the primary site to a local lymph nodes followed by the subsequent spread of one of the progeny of that cell away from the node to the periphery. By measuring the breast cancer death rate among subpopulations of patients sorted by both the size of the primary mass and the number of such positive lymph nodes, we have recently found that the presence of each positive node is associated with an extra 6.08% chance of death (Michaelson *et al*, 2003). It is possible to use this information with equation (2) to measure the probability of the spread of breast cancer cells from the nodes to the periphery (*p*_BC-from-nodes_). To carry out this calculation, we set the value of *L*=0.0608, and to determine the value of *N*, we collected data on the sizes of metastases in lymph nodes, revealing a mean size of 5.3 mm, which is equivalent to *N*=7.84 × 10^6^ cells (Table 3). It follows with equation (2) that the probability of the spread of cancer cells from lymph nodes *p*_BC-from-nodes_=7.96 × 10^−09^≈1 event of spread for every 10^8^ cells. This value is remarkably close to the value for the probability of the lethal spread of breast cancer cells from the primary mass in the breast when the primary mass is also 5.3 mm (*p*_BC-overall_=7.49 × 10^−09^), as calculated by extrapolation of equation (3). This reveals that the occurrence of an event of spread of cancer cells from the primary site in the breast to the local lymph nodes does not appreciably change the tendency of the progeny of those cancer cells to make yet a second event of spread from the lymph nodes to the periphery.

## DISCUSSION

It has often been wondered whether mutation at the time of spread is a requirement for metastasis (Cifone and Fidler, 1981; Fidler, 1983; Sobel, 1990; Welch *et al*, 2000; Yokota, 2000; Bernards and Weinberg, 2002; Couzin, 2003; Bernards, 2003), but the values of the probabilities of metastatic spread of breast cancer and melanoma cells revealed by equation (2) are difficult to reconcile with such genetic changes due to several reasons: *First*, the value of the probability of spread for the smallest melanomas (0.1 mm), at ∼1 event of spread for every 500 cells, is many orders of magnitude greater than that expected for a genetic change. *Second*, the occurrence of one event of spread (the spread of breast cancer cells from the breast to the local lymph nodes) does not appear to increase the chance of a second event of spread (the spread of breast cancer cells from the local lymph nodes to the periphery). In other words, the occurrence of the initial event of spread does not lead to a cell-heritable change in the tendency of the progeny of that cell to spread. *Third*, the data shown here reveal that the probability of metastatic spread per cell declines as tumours increase in size. While this decline is consistent with a number of explanations that are mechanical, (using this term in the sense in which it is used in physics: ‘pertaining to the relations of force and matter’), such as the effect of tumour geometry on the ease of the escape of cells from the primary mass (see Supplementary material), it is not what would be expected for genetic events. Indeed, the probability of genetic events over time should be expected either to remain constant (if only a single genetic event is required) or to increase with time (if the accumulation of multiple genetic events is required). Taken together, these findings would appear to be in agreement with the viewpoint put forward by Bernards and Weinberg ‘that the tendency to metastasise is largely determined by the identities of mutant alleles that are acquired relatively early during multistep tumorigenesis’, and that ‘genes and genetic changes specifically and exclusively involved in orchestrating the process of metastasis do not exist’ (Bernards and Weinberg, 2002).

## Figures and Tables

**Table 1:** Data and calculations of the probability of the spread of cancer cells

**Cancer**	**Population**	**Size range**	**Nominal tumour size (mm)**	**Cell number (*N*)**	**Manifestation of metasatasis**	**Fraction of patients with a manifestation of metastasis (*L*) (%)**	**Probability of spread (*p*=−ln(1−*L*)/*N*)**
Breast cancer	Tabar *et al*	10–14	12	9.05E+07	Cancer death	13	1/6.5 × 10^8^
Breast cancer	Tabar *et al*	15–19	17	2.57E+08	Cancer death	20	1/1.2 × 10^9^
Breast cancer	Tabar *et al*	20–29	25	8.18E+08	Cancer death	45	1/1.4 × 10^9^
Breast cancer	Tabar *et al*	30–49	39	3.11E+09	Cancer death	56	1/3.9 × 10^9^
Breast cancer	Tubiana *et al*	10–25	17	2.57E+08	Cancer death	27	1/8.2 × 10^8^
Breast cancer	Tubiana *et al*	26–35	30	1.41E+09	Cancer death	42	1/2.6 × 10^9^
Breast cancer	Tubiana *et al*	36–45	40	3.35E+09	Cancer death	55	1/4.1 × 10^9^
Breast cancer	Tubiana *et al*	46–55	50	6.54E+09	Cancer death	66	1/6.1 × 10^9^
Breast cancer	Tubiana *et al*	56–65	60	1.13E+10	Cancer death	78	1/7.4 × 10^9^
Breast cancer	Tubiana *et al*	66–75	70	1.80E+10	Cancer death	83	1/1.0 × 10^10^
Breast cancer	Tubiana *et al*	76–85	80	2.68E+10	Cancer death	81	1/1.6 × 10^10^
Breast cancer	Tubiana *et al*	86–95	90	3.82E+10	Cancer death	92	1/1.5 × 10^10^
Breast cancer	Van nuys	10–14	12	9.05E+07	Cancer death	14	1/6.0 × 10^8^
Breast cancer	Van nuys	15–19	17	2.57E+08	Cancer death	28	1/7.8 × 10^8^
Breast cancer	Van nuys	20–29	25	8.18E+08	Cancer death	33	1/2.0 × 10^9^
Breast cancer	Van nuys	30–49	39	3.11E+09	Cancer death	46	1/4.0 × 10^9^
Breast cancer	Van nuys	15–19	17	2.57E+08	Cancer in nodes	32	1/6.7 × 10^8^
Breast cancer	Van nuys	20–24	22	5.58E+08	Cancer in nodes	39	1/1.1 × 10^9^
Breast cancer	Van nuys	25–29	27	1.03E+09	Cancer in nodes	39	1/2.1 × 10^9^
Breast cancer	Van nuys	30–34	32	1.72E+09	Cancer in nodes	50	1/2.5 × 10^9^
Breast cancer	Van nuys	35–39	37	2.65E+09	Cancer in nodes	54	1/3.4 × 10^9^
Breast cancer	Van nuys	40–44	42	3.88E+09	Cancer in nodes	63	1/3.9 × 10^9^
Breast cancer	Van nuys	45–50	47	5.44E+09	Cancer in nodes	54	1/7.1 × 10^9^
Breast cancer	Van nuys	50–54	52	7.36E+09	Cancer in nodes	59	1/8.3 × 10^9^
Melanoma	Balch *et al*	—	0.10	5.3E+01	Cancer death	11	1/480
Melanoma	Balch *et al*	—	0.23	6.8E+02	Cancer death	9	1/7100
Melanoma	Balch *et al*	—	0.37	2.6E+03	Cancer death	10	1/2.4 × 10^4^
Melanoma	Balch *et al*	—	0.44	4.3E+03	Cancer death	14	1/2.9 × 10^4^
Melanoma	Balch *et al*	—	0.50	6.7E+03	Cancer death	18	1/3.4 × 10^4^
Melanoma	Balch *et al*	—	0.60	1.2E+04	Cancer death	22	1/4.5 × 10^4^
Melanoma	Balch *et al*	—	0.70	1.8E+04	Cancer death	18	1/9.1 × 10^4^
Melanoma	Balch *et al*	—	0.84	3.1E+04	Cancer death	20	1/1.4 × 10^5^
Melanoma	Balch *et al*	—	0.91	3.9E+04	Cancer death	23	1/1.5 × 10^5^
Melanoma	Balch *et al*	—	0.97	4.8E+04	Cancer death	21	1/2.0 × 10^5^
Melanoma	Balch *et al*	—	1.11	7.1E+04	Cancer death	20	1/3.1 × 10^5^
Melanoma	Balch *et al*	—	1.17	8.5E+04	Cancer death	27	1/2.7 × 10^5^
Melanoma	Balch *et al*	—	1.31	1.2E+05	Cancer death	30	1/3.3 × 10^5^
Melanoma	Balch *et al*	—	1.38	1.4E+05	Cancer death	25	1/4.8 × 10^5^
Melanoma	Balch *et al*	—	1.51	1.8E+05	Cancer death	30	1/5.0 × 10^5^
Melanoma	Balch *et al*	—	1.58	2.1E+05	Cancer death	25	1/7.1 × 10^5^
Melanoma	Balch *et al*	—	1.71	2.6E+05	Cancer death	30	1/7.1 × 10^5^
Melanoma	Balch *et al*	—	1.85	3.3E+05	Cancer death	32	1/8.3 × 10^5^
Melanoma	Balch *et al*	—	1.88	3.5E+05	Cancer death	38	1/7.1 × 10^5^
Melanoma	Balch *et al*	—	1.98	4.1E+05	Cancer death	44	1/7.1 × 10^5^
Melanoma	Balch *et al*	—	2.11	4.9E+05	Cancer death	40	1/1.0 × 10^6^
Melanoma	Balch *et al*	—	2.25	6.0E+05	Cancer death	45	1/1.0 × 10^6^
Melanoma	Balch *et al*	—	2.52	8.3E+05	Cancer death	36	1/1.9 × 10^6^
Melanoma	Balch *et al*	—	2.72	1.1E+06	Cancer death	45	1/1.8 × 10^6^
Melanoma	Balch *et al*	—	3.02	1.4E+06	Cancer death	47	1/2.3 × 10^6^
Melanoma	Balch *et al*	—	3.39	2.0E+06	Cancer death	50	1/2.9 × 10^6^
Melanoma	Balch *et al*	—	3.72	2.7E+06	Cancer death	54	1/3.4 × 10^6^
Melanoma	Balch *et al*	—	4.26	4.1E+06	Cancer death	54	1/5.3 × 10^6^
Melanoma	Balch *et al*	—	4.73	5.5E+06	Cancer death	55	1/7.1 × 10^6^
Melanoma	Balch *et al*	—	5.27	7.7E+06	Cancer death	59	1/8.3 × 10^6^
Melanoma	Balch *et al*	—	5.77	1.0E+07	Cancer death	57	1/1.2 × 10^7^
Melanoma	Balch *et al*	—	6.74	1.6E+07	Cancer death	63	1/1.6 × 10^7^
Melanoma	Balch *et al*	—	7.75	2.4E+07	Cancer death	65	1/2.3 × 10^7^
Melanoma	Balch *et al*	—	12.32	9.8E+07	Cancer death	76	1/6.7 × 10^7^

**Table 2:** The values of the probabilities of various events of cancer spread

**Metastatic event**	**Probability of spread per cell *p*=-ln(1-*L*)/*N***	**Source of information for the value of *L***	**Value of *p* the probability of spread per cell for tumour masses of ∼5 mm**	**Nature of relationship between the value of *p* and tumour size**
Lethal spread of breast cancer from the primary site in the breast to periphery, pathway unknown	*p*_BC-overall_	*L*_BC-overall_=the fraction of breast cancer deaths among all patients	8.27 × 10^−9^	*p*=*aN*^*b*^ * a*_BC-overall_*≈0.000056 b*_BC-overall_*≈-0.56203*
Lethal spread of melanoma from the primary site in the skin to periphery, pathway unknown	*p*_MEL-overall_	*L*_MEL-overall_=the fraction of melanoma deaths among all patients	1.23 × 10^−7^	*p*=*aN*^*b*^* a*_MEL-overall_*≈0.027 b*_MEL-overall_*≈-0.7836*
Nonlethal spread of breast cancer from the primary site in the breast to the lymph nodes	*p*_BC-to-nodes_	*L*_BC-to-nodes_=the fraction of node positive patients among all patients	1.75 × 10^−8^	*p*=*aN*^*b*^ * a*_BC-to-nodes_*≈0.000092 b*_BC-to-nodes_*≈-0.69251*
Lethal spread of breast cancer from the lymph nodes to the periphery	*p*_BC-from-nodes_	*L*_BC-from-nodes_=6.08%, the lethal contribution per positive lymph node	7.96 × 10^−9a^	Undefined

a The size of nodal metastases was found to have a mean value of 5.3 mm (Table 3). The value shown here for *p*_BC-overall_ is for a mass of 5 mm; for 5.3 mm, *p*_BC-overall_=7.49 × 10^−09^.

**Table 3:** Sizes of the invasive breast cancer metastases seen in the lymph nodes

**Node number**	**Patient number**	**Diameter (mm)**	**Fraction of the metastatic area containing cancer (%)**	**Diameter (corrected)**
49	16	0.3	100	0.3
32	9	1	100	1
20	3	1	100	1
31	8	1.1	100	1.1
43	14	1.4	100	1.4
41	14	1.9	65	1.5
17	2	2	100	2
21	4	2	95	2
39	12	2.1	100	2.1
13	2	2.5	93	2.4
29	7	2.7	95	2.6
15	2	3	95	2.9
30	7	3	100	3
36	12	3	95	3
2	1	3.2	10	3.2
22	5	3.4	95	3.3
5	1	3.4	93	3.3
34	10	3.7	95	3.7
37	12	3.9	100	3.9
4	1	3.9	86	3.6
47	15	4.3	95	4.2
35	11	4.5	95	4.4
40	13	5.4	95	5.2
6	1	5.4	86	5
16	2	5.4	100	5.4
23	5	5.5	76	4.8
33	10	5.5	78	4.9
24	5	6	80	5.4
48	15	6	90	5.7
42	14	6.4	10	6.4
45	14	7.1	60	5.5
10	2	7.1	100	7.1
11	2	7.1	100	7.1
12	2	7.7	95	7.5
18	2	8	43	5.2
19	2	9	72	7.6
46	14	9	90	8.5
28	6	9	80	8
1	1	9.8	84	9
44	14	10	49	7
9	2	10	65	8.1
3	1	10	86	9.3
14	2	10	95	9.7
7	1	10.7	55	7.9
26	6	11	35	6.5
8	2	11	95	10.7
25	6	12.5	45	8.4
27	6	14.5	85	13.4
38	12	15	100	15
Average		5.95	86	5.31
