# Supplementary material for: Spread of human cancer cells occurs with probabilities indicative of a nongenetic mechanism
Source: Br J Cancer. 2005 Nov 8;93(11):1244–9. doi: 10.1038/sj.bjc.6602848 (PMC2361524; doi:10.1038/sj.bjc.6602848)
Supplement: Supplementary Material [file 93-6602848x1.doc]

SUPPLEMENTARY MATERIAL

Some possible mechanisms for why the per-cell probably of spread declines with tumor size

One of the striking findings of the calculations described above is that when data were analyzed for the tumors of various sizes, the value of the probability of spread per-cell appeared to decline in a very characteristic fashion, such that this decline is closely fit by a power function of the form of eq. (3). There are several possible explanations for why this decline might occur.

Explanations arising from cellular heterogeneity

As we noted at the outset, while we have defined *p* in terms of the probability of spread per-cell, we do not assume that every cell in a tumor has the capacity to spread. Indeed, such heterogeneity could be one explanation for why the probability per-cell in the tumor as a whole declines as tumors increase in size. Consider the case in which there are two kinds of cells in a tumor, those that have the capacity to spread to the periphery, with a constant probability *p_s_.*, and those that do have the capacity to spread to the periphery, with the probability *p_u_*=0. Let us first imagine that cells start out having this capacity to spread, but can lose it in a cell heritable fashion. Let us further imagine that in each cell division there is a chance (*p_l_)* that the dividing cell and its progeny irreversibly loses the capacity to spread to the periphery. There is, then, a subpopulation of cells that have the ability to form distant lethal metastases described by:

$N_{s}=N(1-p_{l})^{n}$ (5)

where *N_s_* is the number of cells that retain the ability to spread in each cell division, *n* is the number of cell divisions, and *N* is the total number of tumor cells. Assuming simple exponential growth of the tumor from one cell, we have:

$n=\ln(N)/\ln(2)$ (6)

Again, for reasons well known to us, if *L* is the fraction of patients dying from breast cancer:

$\ln(1-L)=-N_{s}p_{s}$ (7)

and, because this subpopulation of cells comprises the only set of cells in the tumor that have the capacity to form distant lethal metastases, we also have:

$\ln(1-L)=-Np$ (8)

where *p* is the probability per-cell of distant lethal metastatic spread for the tumor as a whole. Combining equations (5)-(8) and solving for *p* yields:

$p=p_{s}(1-p_{l})^{\ln N/\ln2}$ (9)

A simple rearrangement of (9) gives:

$p=p_{s}N^{\ln(1-p_{l})/\ln2}$ (10)

or:

*p = aN ^b^* (4)

for *a = p_s_* and *b = ln(1-p_l_)/ln2.* For example, let us take *p_l_* = 1/3. *p_l_* would take on such a value if in each cell division there was a 1-in-3 chance that the dividing cell and its progeny irreversibly lost their capacity to spread to the periphery. It follows that the value of *b* will be –0.58, which is nearly exactly that given by empirical data. Both the form of the expression derived from this model, then, and the N-dependency of the expression, which is given by the parameter *b,* closely approximate the expression for the probability per-cell of distant lethal metastatic spread as derived from empirical data; the empirically derived relationship is given by a similar power function, with a value of *b=‑0.5611*.

Explanations arising from tumor geometry

Another category of explanations arises from the simple geometrical barriers to the escape of cells from tumor masses, which become more formidable as size increases (Figure 3). Recall that as a sphere increases in size, neither its surface area, nor its diameter, will increase as much as its volume. If these are the places from which cancer cells leave the mass, then their escape will become progressively more difficult as tumor size increases, and the probability of escape per-cell in the mass as a whole will decline. Let us examine the consequences of such geometrical constraints.

Geometrical model #1

Consider *N* to be the number of cells in the tumor of radius *r* where *s* is the density of cells per cc. Thus:

$N=s(4/3)\pi r^{3}$ (11)

Let *N_s_* be the number of cells in a subpopulation of cells that are capable of spreading. Let us further consider the case shown in Figure C above, where the only cells with this capacity are those cells that are next to the lymph duct. If the cells each have a diameter of *d*, and *c* is the number of cells that form a ring around the lymph duct, which is of constant diameter, it follows that the number of cells next to the lymph duct is:

$N_{s}=cr/d$ (12)

Combination with (11) yields:

$N_{s}=N^{1/3}q$ (13)

where:

$q=c/[d(4/3s\pi)^{1/3}]$ (14)

Let *p_s_*, a constant, be the probability of an event of spread per-cell for *N_s_* cells in the subpopulation of cells. Let *p*, a variable, be the probability of an event of spread, per-cell in the tumor as a whole, comprised of *N* cells. Let *L* be the fraction of patients dying of breast cancer. For reasons well known to us:

$1-L=e^{-p_{s}N_{s}}$ (15)

and, because we are assuming that only the cells in the subpopulation immediately adjacent to the lymph duct can give rise to lethal metastatic spread:

$1-L=pN$ (16)

thus

$-\ln(1-L)=p_{s}N_{s}$ (17)

and

$-\ln(1-L)=pN$ (18)

setting (17) and (18) equal to one another and rearranging yields:

$p=p_{s}N_{s}/N$ (19)

combining (13) and (19) yields:

$p=qN^{0.333}/N$ (20)

or

*p = aN ^b^* (3)

where *a=q* p_s_* and *b=-0.666*. Thus, simple geometrical constraints to the spread of cancer cells can give rise to a probability of spread per-cell that declines as tumors increase in size in such a way that this decline conforms to a power function of the form of eq. (3), the N-dependence of which is close to the value found empirically.

Geometrical model #2

We have also been able to show that a simple geometrical model for lethal metastatic spread through the lymphatic system based on a random walk closely approximates the value of *b*, the empirically derived parameter describing the N-dependency of the probability of spread per-cell. In this model, we assume that each cell is equally likely to take a step in any one of the six possible spatial directions, and that if a series of steps results in the cell arriving at a lymph duct, the cell then has a constant intrinsic probability of lethal spread through the lymphatic system. Thus, the probability per-cell of lethal metastatic spread is defined by two factors: the simple geometrical problem of arriving at the lymph duct, and a constant probability that it will spread once it has reached the lymph duct. Using an unbounded random walk model for tumor cells that yields a three-dimensional Gaussian distribution of the probable locations of each cell in the tumor, and assuming that the primary route of spread is through a lymphatic duct modeled as a single point on the surface of the tumor, we were able to arrive at a probability per-cell of lethal metastatic spread through the lymphatic system given, as a function of time and position, by:

$P_{t}=(1/4\pi t)^{3/2}e^{r^{2}/4}r\sin(\theta)drd\theta d\varphi$ (21)

where r, θ, and φ are canonical spherical coordinates. Integrating over all cells in the tumor and until the time of spread, and dividing by the number of cells in the tumor yields an average probability per-cell of lethal spread for the tumor as a whole:

$\left\langle P\right\rangle=1/N\iiint(1/4\pi t)^{3/2}e^{[D(\theta,\varphi,r)]^{2}/4t}r\sin(\theta)drd\theta d\varphi$ (22)

where φ ∈ [0, 2π] , θ ∈ [0, π] , r ∈ [0, (N/C)^1/3^] and D (θ, φ, r) is the distance from any point (θ, φ, r) to the lymph duct, as given in spherical coordinates and assuming a constant cell density of 10^8^ cells/cc:

$D(\theta,\varphi,r)=[(r\sin\theta\cos\varphi)^{2}+(r\sin\theta\sin\varphi)^{2}+(r\cos\theta-0.0062N^{1/3})^{2}]^{1/2}$ (23)

We were unable to find a closed-form solution to integral (22), but numerical integration and graphical analysis reveals that the points generated are very well fit (R^2^ > 0.99) by an equation of the form (data not shown):

*p = aN ^b^* (3)

where the value of b is -0.5562, which is almost exactly that given by empirical data: b = -0.5611.

**Testing these possibilities**

While the modeling described above reveals that the explanations of cell heterogeneity and geometry are possible explanations, they tell us nothing about whether they are the actual explanations. However, these possibilities should be testable in experimental systems with transplantable tumors that give rise to metastases if the relationship between tumor size and the fraction of animals with signs of metastasis are such that the probability of spread per-cell conforms to eq. (3), with the parameter *b* lying in the range ~ -0.5 to -0.8. The hypothesis of cell heterogeneity would be born out by disaggregating tumors of various sizes, injecting the cells intravenously, and determining if the number of metastatic colonies created agreed with that found for the intact tumors of various sizes. The hypothesis of geometry could be tested in a variety of ways in which the geometry of the tumor mass was manipulated. For example, eq. (3) leads to the prediction that the number of metastatic colonies found in two animals containing the same mass of the same tumor will not be same if the tumor is present in one animal as a single transplanted mass (for example in a single limb) while present in the second animal as four smaller masses (for example, by transplanting into each of four limbs), each mass of which is one-fourth of the mass in the animal with a single transplanted tumor. A variety of other manipulations, such as growing the transplanted tumor between glass plates, would alter the geometry of the tumor, and thus might be expected to affect the relationship between tumor size and the probability of spread per-cell.
